# Everybody's Page

**Published:** 1888-12-29

**Authors:** 


### Everybody's Page.

There were between thirty and forty cases of poisoning lately in Rendsburg, Germany, which, when inquired into, proved to be due to drinking artificially-made mineral water.

Dr. Ollivier thinks that children suspected of suffering from measles should be isolated before the eruption appears. The disease is contagious from the moment of its onset, which is usually four or five days before the eruption appears.

Mental overwork is as fruitful a cause of dyspepsia as any, and the effects of worry and anxiety are similar. Grief or fear take away appetite, and a sudden mental shock has been known to cause jaundice.

It has been suggested that as cold is one of the conditions of recovery in yellow fever, the methods employed in the artificial manufacture of ice should be applied to reducing the temperature of yellow fever wards to nearly freezing point, which, it is believed, would do much to lessen the mortality.

The ideal life has been expressed in the following quatrain.

Eight hours to work,
Eight hours to play,
Eight hours to sleep,
And eight shillings a day.

It is generally thought that the use of the eyes in any trade that requires close study of details is injurious to the sight; but it has been proved by practical experience that the sight of watchmakers is quite as good as that of other men, if not better.

In prolonged illnesses it is advisable, whenever it is practicable, to have two beds in the sick-room, and change the patient from one to the other twice in the twenty-four hours, or so. Often the change will bring needed sleep when other means have failed.

Ophthalmia is terribly common in the Russian army. In the hospitals of the Odessa district alone there have been as many as two thousand six hundred sufferers from this cause at one time. But the doctors don't trouble about it, and the nurses treat the cases as they can.

Valuable as public baths are there are certain disadvantages connected with their use. Most people take their "weekly wash" early in the day, and having had the pores of their skin opened by the action of hot water they are apt to catch a chill, either on their way home or in the perform ance of their subsequent duties.

Those extreme teetotallers who boast that they will not take alcohol even when ordered by the doctor, should remember that alcohol is a powerful medicine, not to be refused any more than quinine. Cases have been known where permanent or fatal weakness of the heart has resulted from a refusal to take the stimulant ordered by a medical man.

In 1537, the Corporation of London obtained possession of a house in Bishopsgate-street, and set it apart for the housing of the insane. This was the first Bethlehem Hospital, subsequently too well-known to fame as Bedlam. In 1675, the institution was removed to Moorfields, and in 1814 to St. George's Fields.

The habit, not unknown even in circles that pretend to the possession of commonsense education, of passing on a prescription on the chance that what did good to Tom will suit Dick and Harry, has been aptly condemned by the doctor who said that such a practice was like two or more people having but one set of false teeth among them.

We must go abroad to learn home news. The American *Medical Record* tells the sad case of a certain doctor in Camden Town who was so hard pressed by the competition of other practitioners that he could not get more than twopence-halfpenny a week from his patients. If this is true, it is disgraceful; but we hope that in this case rumour and travel have magnified the hardships of practice in Camden Town.

Among the poor a popular remedy for rheumatism and other aches and pains is a piece of new flannel applied to the part affected. If one inquires why new flannel is selected, the explanation given will probably be that there is a mysterious "oil" in new flannel which is good for the pain; but as a matter of fact the merit seems to consist in the power of irritating the skin which new flannel possesses in a greater degree than old. It is in such cases a mild counter-irritant.

A doctor's wife recently told her husband that he was pleasant everywhere but at home, that in short he was "a house deil and a causeway saint," as the Scotch put it. "My dear," replied the wearied and worried practitioner, "I go out every morning with an immense amount of good temper, but my patients require so much of it that the stock is exhausted before I get home." Dear patients, don't demand too much of your doctor; pity his poor wife on whom falls the brunt of your fretfulness and exactions.

It has long been thought that the two hemispheres of the brain were twins, acting simultaneously and in unison. The opinion of M. J. Luys is different. He believes that each hemisphere is capable, in its normal state, of a certain amount of independent action, which is shown in many things, such as playing the piano, where the two hands touch different notes. To this cause, also, he sets down certain forms of madness, where the patients are sane on all points save one. This is caused, he imagines, by disease of one hemisphere, which has not yet infected the other. When both are diseased confirmed idiotcy or complete insanity generally results.

"Are men dying out?" asks an American Professor, Dr. Sanford S. Chaille. The average life of woman is longer than that of man, and at equal years woman's expectation of life is greater. But by way of compensation, more males are born than females, and of late years the disparity between the average life of the two sexes has been disappearing, perhaps because women are betaking themselves more to those active employments which expose them to accident, wind, and weather. In fact, most American insurance tables are in favourof men in all but sixteen of the years from ten to ninety-nine, so that while this is becoming more and more a woman's world, there is a proportionately less chance of its becoming a world of women.

Dr. Mary Durand has been appealing to the women of France to become army nurses. There are already several associations of women to render aid to the injured: the Red Cross, the Ladies of France, the Union of Frenchwomen, numbering in all about six thousand members; but Dr. Durand thinks there is room for yet a fourth association, ready to tend the sick and wounded in times of war or peace. The appeal is made in stirring terms, and after having done full justice to both the tenderness and the courage of women, Dr. Durand concludes: "If ever the clarion of war should sound, young or old, Catholics or freethinkers, Monarchists or Republicans, we will follow your flag, that of charity, because we have all the love of our country in our hearts."

